# Commentary

**DOI:** 10.4103/0970-0358.53009

**Published:** 2009

**Authors:** S. S. Chatterjee

**Affiliations:** Department of Plastic Surgery, Institute of Post Graduate Medical Education and Research, Kolkata, India

The authors have taken up a difficult subject and a group of patients who are usually neglected both by family members and medical community. Their idea was to create a road map for dealing with patients of lymphedema in an endemic zone. While an attempt has been made keeping the existing socio-economic status and availability of resources in mind, which is commendable, yet from a scientific standpoint, attention to certain points enumerated below would have improved the quality of the paper.

1. This study reports only initial results. The disease is a chronic one and therefore short term results do not mean much. Prevention of recurrent adenolymphangitis (ADL) is probably the most important in the treatment protocol of these patients. Pneumatic compression helps in prevention of such attacks. Ancillary measures are also of equal importance if not more as admitted by authors. The relative importance of each can be discerned if one group of patients has only pneumatic compression and the other only ancillary measures for prevention of attacks.
2. There is no mention of follow up either. Since it was a state sponsored programme, village health workers could have been employed to undertake follow up. In our country lack of follow up may mean poor result as well.
3. The authors have theorized the reason behind effectiveness of the compression therapy which is interesting but there has been no attempt at scientific verification. The theory of generation of heat as a result of sustained unilocular pressure for a certain period has not been substantiated by measurement. It has also been postulated that macrophage activity increases as a result of increased temperature within the tissues. The duration of maintenance of optimal temperature for macrophages to continue activity needs to be determined. At the same time Coumarin (200 mg daily) is added to the regime. Coumarin causes proteolysis and resorption induced by stimulation of macrophages.[1,2] So, it is difficult to conclude unless the study includes patients without Coumarin. It must also be admitted that ultrasound therapy also has been found to be effective and works by generation of heat.[3,4]
4. The grading of lymphedema was not done. So we are not sure about responses to treatment of various grades. As per the authors' postulation, more the fibrosis, more the heat generation in tissue. As a corollary, the advanced cases should have better results. But this is not the experience.
5. The ancillary treatment common to all groups includes foot care, Coumarin and use of crepe bandage compression. It is reported in literature that simple elastic compression garments cause a 30% to 40% reduction of edema.[5,6]
6. Since it was a State government sponsored programme, an attempt could have been made (atleast for those treated with Intermittent Pneumatic Compression) to obtain some objective data showing the status of lymphatics resulting from the different modes of therapy through lymphoscintigraphy[7,8,9] and MRI.[10,11] A few patients with different grades of lymphedema could be selected and their pre- and post-therapy status compared.

I am sure the authors are dedicated to the subject and will consider these points in due course for future publications with establishment of their theory and objective analysis of investigations.
